# Supplementary material for: Mpox-Related Stigma Among Gay, Bisexual, and Other Men Who Have Sex with Men: A Narrative Review
Source: Healthcare (Basel). 2025 Oct 23;13(21):2690. doi: 10.3390/healthcare13212690 (PMC12607514; doi:10.3390/healthcare13212690)
Supplement: Supplementary file 1 [file healthcare-13-02690-s001.zip › healthcare-3851451-supplementary.pdf]

Supplementary File S1

Search strategies

MEDLINE via Ovid

1. exp Monkeypox virus/
2. mpox.tw,kf.
3. monkeypox.tw,kf.
4. monkey pox.tw,kf.
5. MPXV.tw,kf.
6. or/1-5
7. exp Prejudice/
8. exp Social Stigma/
9. stigma.tw,kf.
10. stigmati*ation.tw,kf.
11. discrimination.tw,kf.
12. social perception.tw,kf.
13. social exclusion.tw,kf.
14. internali*ed stigma.tw,kf.
15. or/7-14
16. exp Homosexuality, Male/
17. men who have sex with men.tw,kf.
18. MSM.tw,kf.
19. gay.tw,kf.
20. bisexual.tw,kf.
21. GBMSM.tw,kf.
22. sexual minority men.tw,kf.
23. queer men.tw,kf.
24. or/16-23
25. 6 and 15 and 24

Embase via Ovid

1. exp monkeypox virus/
2. mpox.tw,kw.
3. monkeypox.tw,kw.

4. monkey pox.tw,kw.
5. MPXV.tw,kw.
6. or/1-5
7. exp stigma/
8. exp social discrimination/
9. exp social perception/
10. stigma.tw,kw.
11. stigmati*ation.tw,kw.
12. discrimination.tw,kw.
13. social perception.tw,kw.
14. social exclusion.tw,kw.
15. internali*ed stigma.tw,kw.
16. or/7-15
17. exp homosexuality/
18. exp bisexuality/
19. exp men who have sex with men/
20. men who have sex with men.tw,kw.
21. MSM.tw,kw.
22. gay.tw,kw.
23. bisexual.tw,kw.
24. GBMSM.tw,kw.
25. sexual minority men.tw,kw.
26. queer men.tw,kw.
27. or/17-26
28. 6 and 16 and 27

CINAHL

S1 (MH "Monkeypox") OR mpox OR monkeypox OR "monkey pox" OR MPXV

S2 (MH "Prejudice") OR (MH "Social Stigma") OR stigma OR stigmati*ation

OR discrimination OR "social perception" OR "social exclusion"

OR "internali*ed stigma"

S3 (MH "Homosexuality, Male") OR (MH "Bisexuality+")

OR (MH "Men Who Have Sex with Men")

OR "men who have sex with men" OR MSM OR gay OR bisexual

OR GBMSM OR "sexual minority men" OR "queer men"

S4 S1 AND S2 AND S3

Web of Science

TS=("mpox" OR "monkeypox" OR "monkey pox" OR "MPXV") AND TS=("stigma" OR "stigmati*ation" OR "discrimination" OR "social perception" OR "social exclusion" OR "internali*ed stigma") AND TS=("men who have sex with men" OR "MSM" OR "gay" OR "bisexual" OR "GBMSM" OR "sexual minority men" OR "queer men")
